# Acute Effects of High-Frequency Insular Stimulation on Interictal Epileptiform Discharge Rates in Patients with Refractory Epilepsy

**DOI:** 10.3390/brainsci12121616

**Published:** 2022-11-25

**Authors:** Thi Phuoc Yen Tran, Antoine Dionne, Denahin Toffa, David Bergeron, Sami Obaid, Manon Robert, Alain Bouthillier, Elie Bou Assi, Dang Khoa Nguyen

**Affiliations:** 1 CHUM Research Center, University of Montreal, Montreal, QC H3T 1J4, Canada; 2 Department of Neurosurgery, Vinmec Central Park International Hospital, Ho Chi Minh City 700000, Vietnam; 3 Division of Neurosurgery, CHUM, University of Montreal, Montreal, QC H3T 1J4, Canada; 4 Division of Neurology, CHUM Research Center, University of Montreal, Montreal, QC H3T 1J4, Canada

**Keywords:** deep brain stimulation, interictal epileptiform discharge frequency, insular epilepsy, high-frequency stimulation

## Abstract

**Rationale**: Deep brain stimulation (DBS) of several sites, such as the thalamus, has been shown to reduce seizure frequency and interictal epileptiform activity in patients with refractory epilepsy. Recent findings have demonstrated that the insula is part of the ‘rich club’ of highly connected brain regions. This pilot study investigated short-term effects of high-frequency (HF) insular DBS on interictal epileptiform discharge (IED) rate in patients with refractory epilepsy. **Methods**: Six patients with drug-resistant epilepsy undergoing an intracranial electroencephalographic study received two sets of 10 min continuous 150 Hz HF-DBS of the insula. For each patient, epileptiform activity was analyzed for a total of 80 min, starting 20 min prior to stimulation set 1 (S1), and ending 20 min after stimulation set 2 (S2). All IEDs were identified and classified according to their anatomic localization by a board-certified epileptologist. The IED rate during the 20 min preceding S1 served as a baseline for comparison with IED rate during S1, S2 and post-stimulation periods. **Results**: HF-DBS of the anterior insula (aINS) was performed in a patient with an aINS epileptic focus (patient 1). HF-DBS of the posterior insula (pINS) was performed in two patients with a pINS epileptic focus (patients 2 and 4), in one patient with an aINS focus (patient 3), and in two non-insular patients (patients 5 and 6). The total IED (irrespective of their location) rate significantly decreased (*p* < 0.01) in two patients (patients 1 and 2) during the stimulation period, whereas it significantly increased (*p* < 0.01) in one patient (patient 6); there was no change in the other three patients. Looking at subsets of spike localization, HF-DBS of the aINS significantly reduced aINS and orbitofrontal IEDs in patient 1 (*p* < 0.01), while HF-DBS of the pINS had an effect on pINS IEDs (*p* < 0.01) in both patients with a pINS focus; there was no significant effect of HF-DBS of the insula on IEDs in temporal or other frontal regions. **Conclusion**: Short-term HF-DBS of the insula had heterogeneous effects on the IED rate. Further work is required to examine factors underlying these heterogeneous effects, such as stimulation frequency, location of IEDs and subregions of the insula stimulated.

## 1. Introduction

Deep brain stimulation (DBS) is proven to be a safe and effective treatment option for patients with pharmacoresistant epilepsy who are not candidates for resective brain surgery [1]. The therapeutic outcome of DBS mainly depends on its target and stimulation parameters, which may account for many conflicting data across different studies. Different stimulation targets have been investigated, including the anterior nucleus of the thalamus (ANT), centromedian nucleus of the thalamus, hippocampus, pulvinar, and cerebellum [1,2]. Only DBS of the ANT has been approved for the treatment of refractory focal epilepsy following a large, double-blind, sham-controlled, and paralleled-group randomized controlled trial [3,4,5]. A small randomized controlled trial and several other non-controlled studies have supported its efficacy [6,7,8,9].

Since the ANT is a vital relay site of the Papez circuit (thought to play an essential role in the generation and propagation of seizures), DBS of the ANT may reduce the cortical excitability or block the spread of focal seizures to cortical areas [1,10]. A recent study in rats showed that high-frequency (HF) DBS of the ANT caused a significant reduction in cell activity in the circuit of Papez, which might help explain its antiepileptic effects [11]. Similarly, the hippocampus (also part of the Papez circuit) has been proposed as a promising target for DBS in the treatment of refractory mesial temporal epilepsy [12,13,14,15,16,17].

Given the rich connections of the insula with other cortical (frontal, temporal, parietal cortices) and subcortical structures (thalamus, putamen, nucleus accumbens, caudate nucleus, and globus pallidus) [18], the insula has recently been proposed as a potential target for DBS in the treatment of refractory neuropathic pain [19], obsessive–compulsive disorder [20], and addiction [21,22]. Considering that the insula is part of the ‘rich-club’ members with dense interconnections, we hypothesized that stimulating it could reduce epileptiform activity locally or in distant connected sites, similar to what is observed with the stimulation of the anterior nucleus of the thalamus. Experimental and human studies verified that HFS can induce the inhibition of cortical excitability and block axonal conduction [23,24,25,26]. In this pilot study, we investigated the potential of insular DBS for epilepsy by assessing if HF stimulation (150 Hz) had any effect on the rate of interictal epileptiform discharges (IED) in patients undergoing an intracranial electroencephalographic (icEEG) study for clinical purposes.

## 2. Materials and Methods

### 2.1. Participants and Surgical Implantation

Patients with drug-resistant epilepsy undergoing an icEEG study with insular sampling were recruited to assess the effect of high-frequency insular stimulation on the rate of IEDs (i.e., number of IEDs per minute). Patients’ characteristics are summarized in Table 1. All subjects were implanted unilaterally in areas of suspected epileptogenicity as determined by the epilepsy surgery team. The method of implantation of insular electrodes was previously described in detail by Surbeck and colleagues [27]. Briefly, after a fronto-temporal craniotomy, the Sylvian fissure was opened and hybrid operculo-insular electrodes [28] or regular depth electrodes (Ad-Tech Medical Instrument Corporation, Racine, WI, USA) were inserted. Depth electrode contacts in the insula each measured 2.4 mm × 1.1 mm and were separated by 5 mm (center to center). A brain MRI was always obtained after implantation to verify the exact position of electrode contacts.

### 2.2. Stimulation Parameters

All patients had two periods of 10 min bipolar insular stimulation using a Grass Stimulator S88 (Grass Instruments, Natick, MA, USA) separated by a one-hour resting-awake interval, except for patient 6 who received one period of 5 min and one period of 10 min stimulation (technical error). HF stimulations (150 Hz) were delivered in bipolar mode by employing the two contacts of the depth part of the hybrid electrode. A 0.75 ms pulse width was maintained in all patients. We sought to use the highest current intensity possible without evoking clinical symptoms (subthreshold stimulation). Hence, thresholds for individual patients were assessed the day prior to DBS experiments: 5 s HF pulses of increasing current intensity were given, starting at 0.5 mA and with increments of 0.5–1 mA until clinical response (e.g., somatosensory, viscerosensory or gustatory evoked symptoms) or a maximum of 10 mA. On the day of DBS experiments, stimulation intensity was set to 70% of the clinical threshold determined on the previous day. Stimulation intensities of 3.3 mA, 0.4 mA, 1.5 mA, and 6 mA were set for patients 1, 2, 3, and 6, respectively. For patients 4 and 5 who did not report any evoked symptoms, stimulation intensity was set to 7 mA. These stimulation intensities did not provoke after-discharges during DBS for all patients.

### 2.3. Data Acquisition and Spike Detection

Patients were awake for at least 1 h before the recording and during all sessions. Antiseizure medications were kept constant throughout the day. All IEDs were identified and marked by a board-certified epileptologist. When a pattern of rhythmic spike and wave activity was encountered, each spike or sharp wave was counted as one IED. When a pattern of spike plus (spike followed by fast activity) was encountered, only the spike component was marked. When a delta brush pattern was found, each delta wave was considered as one IED.

**Figure 1:**
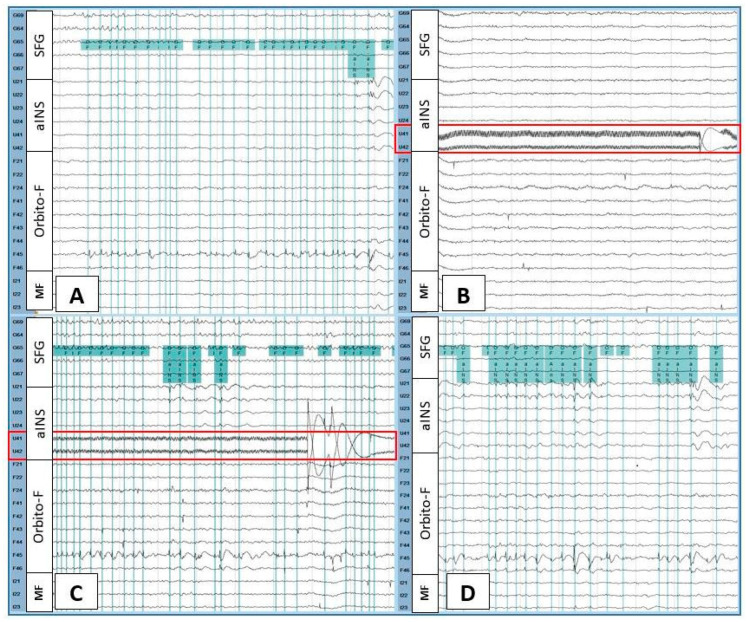
Intracranial EEG recording during baseline (**A**), the beginning of the first stimulation (**B**), the late phase of the first stimulation (**C**), and the first post-stimulation period (**D**) in patient 1. The green vertical lines indicate marked spikes seen at the junction of the orbitofrontal operculum (F45 > F46) and the anterior insula (U21, U22, U23). High-frequency stimulation was performed over U41-U42 anterior insular contacts (red rectangle). At the beginning of the stimulation, orbitofrontal and orbitofrontal operculum–anterior insular spikes disappeared (**B**), then gradually reappeared (**C**). These interictal epileptiform discharges continued abundantly during the post-stimulation period (**D**). Filters (high-pass = 1 Hz, low-pass = 70 Hz) were set only for illustrative purposes. SFG = superior frontal gyrus; aINS = anterior insula; Orbito-F = orbitofrontal; MF = medial frontal.

Each IED type was classified according to its anatomic localization. For example, if the contacts involved in the electrical field of an IED were over the anterior insula (aINS), then that IED was named aINS IED. When two different localizations were involved, both regions were included in the IED name. If three or more regions were involved, we classified this IED type as synchronous IED. We considered the opercular and insular areas as one region. Figure 1 shows an example of the IEDs marking procedure.

For each patient, the interictal epileptiform activities were analyzed for 20 min prior to the first stimulation [baseline (B)], 10 min during the first stimulation (S1), 20 min after S1 (PS1), 10 min during the second stimulation (S2), and 20 min after S2 (PS2). The number of each IED type for each one-minute segment was counted using Brain Vision Analyzer 2.1 commercial software.

### 2.4. Statistical Analysis

All statistical analyses were performed using the statistical package for the social sciences (SPSS) version 27. For each patient, a total of 80 one-minute segments were analyzed, except for patient 6, for whom the first stimulation was only five minutes due to technical problems. IED rate is reported in IEDs per minute. The median and interquartile range (IQR) of each IED rate were calculated and expressed as a median (IQR). Since the data distribution was not normal, a Mann–Whitney rank-sum test was applied to determine the difference in IED rate between B vs. S1, B vs. PS1, B vs. S2, and B vs. PS2. We divided the post-stimulation period into two equal periods: the first ten minutes and the last 10 min to see whether the post-stimulation effects of the HFS changed with the time.

## 3. Results

**Figure 2:**
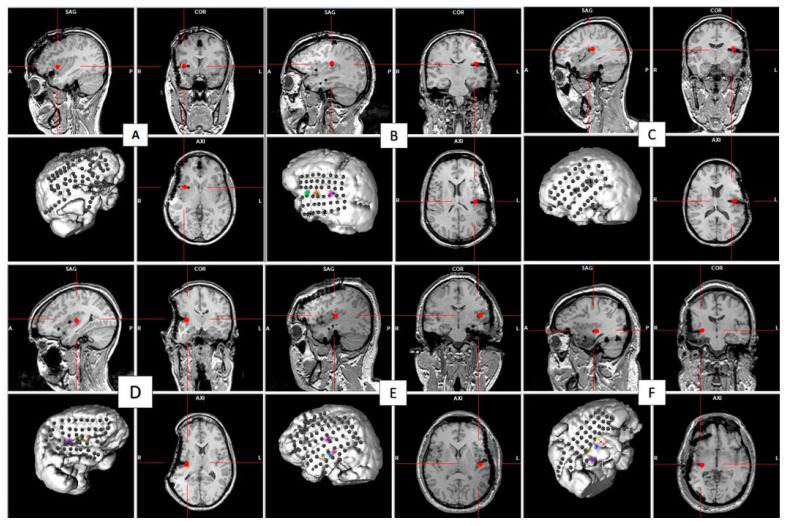
The 3-D representations of each patient’s brain with icEEG recording sites and location of the insular site of stimulation (small red cross) on the sagittal, axial, and coronal T1-weighted MRI cuts. The stimulation site was in the anterior insula in patient 1 (**A**) and in the posterior insula in the others ((**B**–**F**) was for patients 2, 3, 4, 5 and 6 respectively).

**Figure 3:**
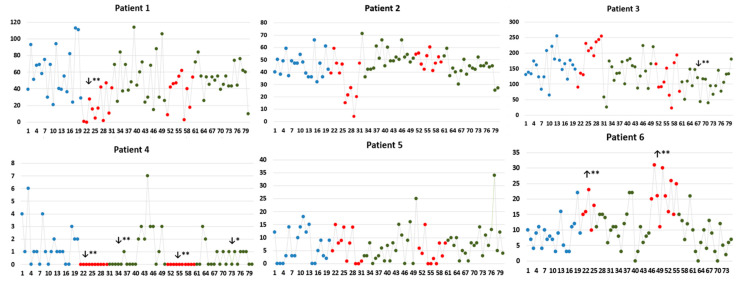
Number of total IEDs (irrespective of localization) every minute during the analyzed periods for each patient. Comparisons were performed between baseline IED rate with the fist stimulation period (S1), post-stimulation period 1 (PS1), the second stimulation period (S2), and post-stimulation period 2 (PS2). ** and * indicate statistical significance at *p* < 0.01 and *p* < 0.05. X-axis: number of IEDs; Y-axis: time (in minutes). Blue, red, and green dots represent IED rates identified during baseline, stimulation and post-stimulation periods, respectively. ↓ and ↑: indicate the significant decrease and increase, respectively, in the IED rate of the studied period compared to that of the baseline.

Figure 2 shows 3-D representations of the patients’ brains, including the recording sites and insular stimulating electrode for each patient. HF-DBS of the aINS (HF-DBS-aINS) was performed in one patient with an aINS epileptic focus (patient 1 = group 1). HF-DBS of the posterior insula (HF-DBS-pINS) was performed in five patients (patients 2–6), two of which had a pINS epileptic focus (patients 2 and 4 = group 2) and one who had an aINS epileptic focus (patient 3 = group 3). Patients 5 and 6 (group 4) did not have insular epilepsy; patient 5 had no insular IEDs, while patient 6 only had rare insular IEDs during invasive EEG monitoring. IED rate changes for each patient are shown in Table 2 and Figure 3.

Group 1: HF-DBS-aINS in Patients with an Anterior Insular Epileptic Focus

This group consisted of one patient (patient 1). During the baseline period, there were abundant IEDs at the junction of the orbito-frontal operculum with the aINS [median (IQR) = 31 (37.75) IEDs/min]. The rate of total IEDs during both stimulation periods was lower than that of the baseline, but only the decrease during the first stimulation period was statistically significant [14 (22.5) vs. 50 (41.5), *p* < 0.01]. There were no significant differences in total IED rates between baseline and post-stimulation periods.

During baseline, the predominant IED type in this patient was located at the junction of the orbito-frontal operculum with the aINS [median (IQR) = 31 (37.75) IEDs/min]. Independent orbitofrontal spikes (i.e., not synchronously involving the aINS) were also observed with a rate of 9 (15.75)/min, and independent aINS IEDs (i.e., not synchronously involving the orbitofrontal EEG contacts) appeared at a rate of 1 (3.75)/min. The first HF-DBS-aINS significantly reduced the rates of all three IED types [0 (2.25) vs. 9 (15.75), *p* < 0.01 for orbitofrontal IEDs; 0 (0.25) vs. 1 (3.75), *p* < 0.05 for aINS; and 7 (21.75) vs. 31 (37.75), *p* < 0.05 for OF-aINS IEDs); however, the second stimulation only significantly reduced the aINS IED rate [0 (0) vs. 1 (3.75), *p* < 0.01]. Notably, there was no independent aINS IED seen during or after the second stimulation period. We also observed an increase in orbitofrontal IED rate after both stimulations, but statistical significance was only met during the first ten minutes after the second stimulation [22 (18) vs. 9 (15.75), *p* < 0.05] and the second ten minutes after the first stimulation [20.50 (16) vs. 9 (15.75), *p* < 0.05].

Group 2: HFS-pINS in Patients with a Posterior Insular Epileptic Focus

In patient 2, there was a significant reduction in operculo-insular IEDs [24 (15.75) vs. 46 (13.75), *p* < 0.01] as well as ‘synchronous IEDs’ [0 (1) vs. 2 (3.75), *p* < 0.05] (i.e., spikes with a larger field of distribution involving not only operculo-insular contacts, but several other icEEG contacts) during the first stimulation; however, only ‘synchronous IEDs’ significantly decreased during the second stimulation [0 (1) vs. 2 (3.75), *p* < 0.05]. We also observed a rebound effect after the stimulation: operculo-insular IEDs increased after the first stimulation, which met statistical significance in the last ten minutes of the first post-stimulation period [51 (7.50) vs. 46 (13.75), *p* < 0.01]; ‘synchronous IEDs’ significantly increased in the first ten minutes of the second post-stimulation period [5 (9.25) vs. 2 (3.75), *p* < 0.05] and then decreased to baseline [(2 (3.25) vs. 2 (3.75)]

As for patient 4, IEDs were relatively rare during the icEEG study. Still, we observed a significant decrease in operculo-insular IEDs during both stimulation periods and the first ten minutes of the post-stimulation period (*p* < 0.001).

Group 3: HFS-pINS in Patients with an Anterior Insular Epileptic Focus

This group consisted of only one patient (patient 3). At baseline, there were abundant aINS IEDs, nearly continuous. Because of this, and to err on the side of caution, it was decided not to stimulate the anterior insula but rather nearby posterior insular contacts. There was no significant reduction in the IED rate during the first stimulation and post-stimulation periods. The second stimulation reduced the rate of aINS IEDs by 40.7% [67 (86.5) vs. 113 (54.75)], but this did not reach statistical significance; however, a significant decrease in the rate of total IEDs was observed during the first ten minutes of the second post-stimulation period (113 (22.75) vs. 154.4 (47), *p* < 0.05).

Group 4: HFS-pINS in Patients without an Insular Epileptic Focus

In patient 5, HF-DBS-pINS did not affect the overall IED rate. There was no significant change after both stimulations. As for more broadly distributed spikes, HF-DBS-pINS significantly decreased the rate of ‘synchronous’ IED only during the second stimulation period [0 (0.75) vs. 1 (6.5), *p* < 0.05]. After the stimulation, we only observed a significant decrease in the lateral temporal IEDs [0 (1.25) vs. 2.5 (4.5), *p* < 0.05] or a significant increase in the lateral prefrontal IEDs [1.5 (2.25) vs. 0 (1), *p* < 0.01] during the first ten minutes of the first post-stimulation period.

In patient 6, the HF-DBS-pINS caused a significant increase in the rate of orbitofrontal IEDs, but there was no effect on the rate of insular IEDs or temporal IEDs.

## 4. Discussion

In this pilot study, we report the acute effects of HF-DBS of the insula on the frequency of IEDs, looking at local insular IEDs, insular IEDs that were more broadly distributed to extra-insular regions, and independent local extra-insular IEDs when present.

Although stimulation of the insula did reduce the rate of local IEDs, insular IEDs that were more broadly distributed, or extra-insular IEDs in some patients, this effect was neither necessarily uniform, consistently reproduced in both stimulation periods, nor sustained in post-stimulation periods. In contrast, an increase in IED rate was even observed in the post-stimulation period in two patients. These heterogeneous results did not appear to be related to the differential connectivity profiles of distinct subinsular areas as IEDs located outside the insula were located in areas known to be connected to the particular subinsular area that was stimulated [18,29]. Hence, at this point, no clear conclusion can be drawn from these heterogeneous findings. However, the fact that an acute reduction in IED rate was sometimes observed warrants further investigation with a much larger number of patients. Only then will we be able to assess if DBS of the insula has any added value compared to existing neurostimulation therapies (vagus nerve stimulation, thalamic DBS, and Neurospace) that have already been validated by large prospective studies.

We acknowledge that our study has several limitations. First, the number of patients is low as we only meant to assess feasibility and obtain preliminary observations to guide a larger study. Second, stimulation current intensities differed across patients; a larger prospective study will allow us to test a few different sets of parameters, which will be kept constant within subgroups of patients [24]. Third, our patients only had a limited number of contacts in the insula to stimulate, preventing us from determining which particular insular subregion(s) is/are more optimal for DBS. Furthermore, considering the technique used to implant electrodes (free-handed insertion of electrodes orthogonally into a thin cortex) and the limitation of using MRI to determine the exact location of the electrode contacts (as contacts appear as ‘black holes’ that extend beyond the radius of individual contacts due to magnetic susceptibility), we acknowledge that it is difficult to be certain what exactly is stimulated (the insular cortex versus the subinsular area, both, or even a larger network). Because our group has since increased the number of electrodes and contacts sampling the insula, notably by stereotactically inserting insular electrodes via a transfrontal or transparietal approach, these considerations should be partially addressed. Fourth, because our patients had unilateral implantations, the effect of bilateral stimulation could not be assessed. Fifth, although high-frequency oscillations are considered reliable biomarkers of epileptogenicity, we did not assess the impact of high-frequency stimulation on their rate; this could be integrated in our future experiments and analyses. Finally, our methodology only allows an assessment of the acute effects of insular stimulation on IED frequency. As more patients with insular epilepsy are implanted with the NeuroPace RNS system, it might be possible to study acute as well as long-term insular stimulation-induced icEEG changes and correlate them with long-term outcomes. In a recent retrospective study, Rønborg and colleagues found that patients with drug-resistant focal epilepsy treated with direct brain-responsive neurostimulation showed an acute stimulation-related reduction in iEEG spectral power, which was associated with reductions in clinical seizure frequency [30]; however, because most patients had mesial temporal lobe epilepsy, it is unlikely that the insula was stimulated in a significant number of patients in the aforementioned study.

## 5. Conclusions

Short-term HF-DBS of the insula had heterogeneous effects on IEDs, including a reduction in IED frequency in some patients. Further work is required to examine factors underlying these heterogeneous effects, such as the stimulation frequency, the location of IEDs, and the subregions of the insula that are being stimulated.

## Figures and Tables

**Table 1:** Patients’ demographic features and stimulation characteristics.

Patient	Age at Onset of Epilepsy/Age at Invasive EEG	Gender/Dominance	Side and Location of Epileptic Focus	Stimulation Intensity (mA)	Location of Stimulated Insular Contacts
1	9/25	Fe/R	R, junction between OF and ant INS	3.3	R aINS
2	16/47	M/R	L, P operculum, pINS	0.4	L pINS
3	5/38	Fe/R	L, F operculum + mid-INS and aINS	1.5	L pINS
4	22/32	Fe/R	R, pINS	7	R INS
5	unknown/25	M/R	L, FT	7	L pINS
6	6 months/41	M/R	R, OF	6	R pINS

Abbreviations: M = male; Fe = female; R = right; L = left; F = frontal; FT = fronto-temporal; P = parietal; INS = insula; OF = orbitofrontal; ant = anterior; mid = middle; p = posterior.

**Table 2:** The rate of IED types before, during, and after the high-frequency stimulation of the insula for each patient.

**Patient 1**	**IED rate (IED/minute); median (IQR)**						
**IED localizations**	**B**	**S1**	**PS1**		**S2**	**PS2**	
			**1** **st 10 min**	**2** **nd 10 min**		**1** **st 10 min**	**2** **nd 10 min**
OF	9 (15.75)	0 (2.25) ↓ (*p* = 0.004)	15.50 (9.75)	20.50 (16) ↑ (*p* = 0.023)	17 (19.17)	22 (18) ↑ (*p* = 0.013)	16.50 (18.50)
aINS	1 (3.75)	0 (0.25) ↓ (*p* = 0.042)	0 (0) ↓ (*p* = 0.003)	0 (0) ↓ (*p* = 0.003)	0 (0) ↓ (*p* = 0.003)	0 (0) ↓ (*p* = 0.003)	0 (0) ↓ (*p* = 0.003)
OF-aINS	31 (37.75)	7 (21.75) ↓ (*p* = 0.025)	35 (38.75)	12.50 (60)	22.5 (7.0)	32.5 (17)	36 (27.25)
**Patient 2**	**IED rate (IED/minute); median (IQR)**						
**IED localizations**	**B**	**S1**	**PS1**		**S2**	**PS2**	
			**1** **st 10 min**	**2** **nd 10 min**		**1** **st 10 min**	**2** **nd 10 min**
T-P operculo-pINS	46 (13.75)	24 (15.75) ↓ (*p* = 0.006)	45 (20.5)	51 (7.50) ↑ (*p* = 0.007)	49 (8.25)	37 (14)	41.50 (6.5)
lateral T	0 (1)	0 (0)	0 (0)	0 (0)	0 (2)	0 (1)	0 (0)
Synchronous	2 (3.75)	0 (1) ↓ (*p* = 0.03)	0 (1) ↓ (*p* = 0.03)	0 (2.25)	0 (1) ↓ (*p* = 0.03)	5 (9.25) ↑ (*p* = 0.038)	2 (3.25)
**Patient 3**	**IED rate (IED/minute); median (IQR)**						
**IED localizations**	**B**	**S1**	**PS1**		**S2**	**PS2**	
			**1** **st 10 min**	**2** **nd 10 min**		**1** **st 10 min**	**2** **nd 10 min**
aINS	113 (54.75)	126 (92.25)	106.50 (8.25)	123.5 (67.26)	67 (86.5)	94 (52.5)	81.5 (60.6)
IFG-aINS	32 (36.5)	60 (40)	20 (20.75)	29.5 (34.75)	29.5 (19)	18 (14.25) ↓ (*p* = 0.022)	22 (21.25)
STG-aINS	0 (4.25)	0 (2)	0 (4.25)	0 (1.75)	0 (1.25)	0 (0)	0 (2.0)
synchronous	1 (11.75)	13 (41)	0 (1)	0 (2)	0 (5.25)	0.5 (3.25)	0 (1.25)
**Patient 4**	**IED rate (IED/minute); median (IQR)**						
**IED localizations**	**B**	**S1**	**PS1**		**S2**	**PS2**	
			**1** **st 10 min**	**2** **nd 10 min**		**1** **st 10 min**	**2** **nd 10 min**
T-P opercula-pINS	1.6 (2.75)	0 (0) ↓ (*p* < 0.001)	0 (0) ↓ (*p* < 0.001)	2 (3.0)	0 (0) ↓ (*p* < 0.001)	0 (1.25)	0.5 (1.0) ↓ (*p* = 0.040)
Synchronous	0 (0)	0 (0)	0 (0)	0 (0.25)	0 (0)	0 (0)	0 (0)
**Patient 5**	**IED rate (IED/minute); median (IQR)**						
**IED localizations**	**B**	**S1**	**PS1**		**S2**	**PS2**	
			**1** **st 10 min**	**2** **nd 10 min**		**1** **st 10 min**	**2** **nd 10 min**
lateral T	2.5 (4.5)	1.5 (7.25)	0 (1.25) ↓ (*p* = 0.010)	3 (5.5)	2.5 (5.75)	2 (4.25)	4.5 (5.0)
lateral prefrontal	0 (1)	0 (0.25)	1.5 (2.25) ↑ (*p* = 0.005)	0.5 (2.25)	0 (1.0)	0.5 (2.0)	0.5 (2.0)
Synchronous	1 (6.5)	0 (1.25)	0 (1.25)	3 (5.5)	0 (0.75) ↓ (*p* = 0.044)	2 (4.25)	1 (4)
**Patient 6**	**IED rate (IED/minute); median (IQR)**						
**IED localizations**	**B**	**S1**	**PS1**		**S2**	**PS2**	
			**1** **st 10 min**	**2** **nd 10 min**		**1** **st 10 min**	**2** **nd 10 min**
OF	6.50 (4.50)	16 (7.0) ↑ (*p* = 0.001)	8 (6.50)	9 (10.75)	21 (11.25) ↑ (*p* < 0.001)	8 (8.25)	5 (5)
pINS	0 (1)	0 (0)	0 (1)	0 (0)	0 (0)	0 (0.25)	0 (0)
Posterior T	1.5 (3)	0 (3)	3.5 (4.25)	1.5 (3.25)	0 (0.75)	0.5 (3.5)	0 (2)

Abbreviations: IED = interictal epileptiform discharge; B = baseline; S1 = first stimulation; S2 = second stimulation; PS1 = first post-stimulation; PS2 = second post-stimulation; aINS = anterior insula; IQR = interquartile range; OF = orbito-frontal; T = temporal; F = frontal; P = parietal; IFG = inferior frontal gyrus; STG = superior temporal gyrus. ↓ and ↑: indicate the significant decrease and increase, respectively, in the IED rate of the studied period compared to that of the baseline.

## Data Availability

The data presented in this study are available on request from the corresponding author. The data are not publicly available due to privacy restrictions.
